# Gene therapy in rare diseases: the benefits and challenges of developing a patient-centric registry for *Strimvelis* in ADA-SCID

**DOI:** 10.1186/s13023-018-0791-9

**Published:** 2018-04-06

**Authors:** Heide Stirnadel-Farrant, Mahesh Kudari, Nadia Garman, Jessica Imrie, Bikramjit Chopra, Stefania Giannelli, Michela Gabaldo, Ambra Corti, Stefano Zancan, Alessandro Aiuti, Maria Pia Cicalese, Rohit Batta, Jonathan Appleby, Mario Davinelli, Pauline Ng

**Affiliations:** 1 GlaxoSmithKline, Stevenage, Hertfordshire UK; 2 GlaxoSmithKline, Brentford, Middlesex UK; 3 GlaxoSmithKline, Upper Merion, PA USA; 4 San Raffaele Telethon Institute for Gene Therapy (SR-TIGET), San Raffaele Scientific Institute, Milan, Italy; 5 Pediatric Immunohematology and Bone Marrow Transplantation Unit, San Raffaele Scientific Institute, Milan, Italy; 6 Vita Salute San Raffaele University, Milan, Italy; 7 Pharmaceuticals Product Development, Milan, Italy

**Keywords:** Adenosine deaminase deficiency, Severe combined immunodeficiency, Gene therapy, Haematopoietic stem cell transplantation, Transplantation, Autologous, Pharmacovigilance

## Abstract

**Background:**

Strimvelis (autologous CD34+ cells transduced to express adenosine deaminase [ADA]) is the first ex vivo stem cell gene therapy approved by the European Medicines Agency (EMA), indicated as a single treatment for patients with ADA-severe combined immunodeficiency (ADA-SCID) who lack a suitable matched related bone marrow donor. Existing primary immunodeficiency registries are tailored to transplantation outcomes and do not capture the breadth of safety and efficacy endpoints required by the EMA for the long-term monitoring of gene therapies. Furthermore, for extended monitoring of Strimvelis, the young age of children treated, small patient numbers, and broad geographic distribution of patients all increase the risk of loss to follow-up before sufficient data have been collected. Establishing individual investigator sites would be impractical and uneconomical owing to the small number of patients from each location receiving Strimvelis.

**Results:**

An observational registry has been established to monitor the safety and effectiveness of Strimvelis in up to 50 patients over a minimum of 15 years. To address the potential challenges highlighted above, data will be collected by a single investigator site at Ospedale San Raffaele (OSR), Milan, Italy, and entered into the registry via a central electronic platform. Patients/families and the patient’s local physician will also be able to submit healthcare information directly to the registry using a uniquely designed electronic platform. Data entry will be monitored by a Gene Therapy Registry Centre (funded by GlaxoSmithKline) who will ensure that necessary information is collected and flows between OSR, the patient/family and the patient’s local healthcare provider.

**Conclusion:**

The Strimvelis registry sets a precedent for the safety monitoring of future gene therapies. A unique, patient-focused design has been implemented to address the challenges of long-term follow-up of patients treated with gene therapy for a rare disease. Strategies to ensure data completeness and patient retention in the registry will help fulfil pharmacovigilance requirements. Collaboration with partners is being sought to expand from a treatment registry into a disease registry. Using practical and cost-efficient approaches, the Strimvelis registry is hoped to encourage further innovation in registry design within orphan drug development.

## Background

Adenosine deaminase (ADA) deficiency is a monogenic disorder of purine metabolism. Accumulation of toxic metabolites of purine nucleotides, normally metabolised by ADA, results in apoptosis in developing lymphocytes, absence of humoral and cellular immune function and severe combined immunodeficiency (SCID) [1]. Diagnosis of SCID is usually made early in life, either through newborn screening (introduced in some states of the USA [2]) or after patients present with severe opportunistic infections, diarrhoea and a failure to thrive [1]. ADA deficiency is an ultra-rare immunodeficiency, with reported incidence rates of between 0.17 and 0.55 per 100,000 live births, translating to less than 50 children per year in the United States (US) and the European Union (EU) [2–7].

ADA-SCID can be fatal within the first year without treatment and requires early intervention. Treatment options include haematopoietic stem cell transplantation (HSCT), enzyme replacement therapy (ERT) and gene therapy. HSCT is potentially corrective for the immunological manifestations of ADA-SCID, but is recommended as first-line treatment only when a matched sibling/family donor (MSD/MFD) is available in order to achieve optimal immune reconstitution and overall survival [8, 9]. Graft versus host disease, a potentially life-threatening complication, occurs more frequently and at greater severity when HSCT is performed with unrelated or less well-matched donors compared with MSD/MFDs [8, 10]. As an alternative, ERT with polyethylene glycol (PEG)-conjugated ADA does not require a compatible bone marrow donor to be available and eliminates toxic purine metabolites without the related complications of transplantation [11]. However, recovery of T-lymphocyte numbers is highly variable between patients, and for those who do respond, T- and B-lymphocyte cell populations remain subnormal at peak levels and may decline after 5 to 12 years of treatment [12, 13]. Complications of ERT may also arise from the development of autoantibodies to ADA, chronic pulmonary insufficiency and lymphoproliferative disorders, resulting in an overall 20-year survival rate of 78% [14]. The requirement for continued once- or twice-weekly injections makes long-term ERT costly and burdensome for patients and their families. Access to treatment can also vary; PEG-ADA is US Food and Drug Administration approved, but is estricted to compassionate use within countries of the EU [14].

ADA-SCID was the first primary immunodeficiency to be genetically characterised, and one of the earliest targets for gene therapy [15, 16]. In May 2016, Strimvelis (autologous CD34+ cells transduced to express ADA; GlaxoSmithKline) was the first ex vivo gene therapy approved for use by the European Medicines Agency (EMA), indicated for the treatment of patients with ADA-SCID for whom no suitable matched-related stem cell donor is available [17, 18]. The approval of gene therapy for ADA-SCID represents a unique step forward in the management of this ultra-rare disease, with the potential to be a one-off corrective treatment of the immunological manifestations for eligible patients. The European Society for Immunodeficiencies (ESID) and the European Society for Blood and Marrow Transplantation (EBMT) have since updated their joint guidelines to recommend gene therapy as first-line treatment for patients with ADA-SCID with no MSD/MFD [9].

As part of the clinical development programme, the safety and efficacy of Strimvelis (known as GSK2696273 prior to marketing authorisation) was evaluated in 18 patients who lacked a MSD/MFD and for whom PEG-ADA ERT was not a viable therapeutic option (i.e. due to intolerance, allergy, autoimmune manifestations, or availability). All 18 patients were alive at the data cut-off for regulatory submission (8 May, 2014) and 14/17 (82%) patients remained intervention-free (i.e. did not require HSCT or ERT for ≥3 consecutive months) after a median (range) follow-up of 6.9 years (2.3 to 13.4 years) [19, 20]. Additional patients have been treated with Strimvelis since 2014 as part of an investigator-initiated Named Patient Programme for compassionate use and, since receiving marketing authorisation, as a commercial product. Patients who received Strimvelis during the clinical development programme were invited to participate in a follow-up study designed to monitor the safety and efficacy of treatment up to 8 years post-gene therapy [21]. As of February 2016, all subjects were alive after a median follow-up of 8.2 years, with over 15 years of follow-up data available for the first subject to undergo treatment [22].

Importantly, no events indicative of leukaemic transformation or myelodysplasia have been reported in patients treated with Strimvelis. Indeed, there have been no cases of lymphoproliferative disease reported in any gene therapy studies for ADA-SCID involving more than 70 patients and using a variety of vectors and treatment approaches [19, 20, 23–30]. In contrast, leukaemia has been reported during clinical trials of gammaretrovirus-based gene therapy for X-linked SCID (X-SCID), chronic granulomatous disease (CGD) and Wiskott−Aldrich syndrome (WAS). These included 6 cases of T-cell acute lymphoblastic leukaemia (T-ALL) in 20 patients affected by X-SCID, [31–33] 7 cases of T-ALL in 10 patients with WAS, [34] and 3 cases of myelodysplastic syndrome in 12 patients treated for CGD [35–37]. With the exception of one case of T-ALL in a patient with X-SCID, which occurred 15 years post treatment, oncogenic events were reported within 6 years after gene therapy. For some primary immunodeficiencies, it is recognised that the pathology of the disease itself may contribute to the overall risk of oncogenesis in patients undergoing gene therapy [38–40].

The EMA has published guidelines on long-term safety monitoring of patients administered advanced therapy medicinal products (ATMPs), such as gene therapy, including assessment of endpoints such as auto-immunity, malignancies and the potential for vector reactivation [41]. Gene therapies may be subject to further post-approval safety and effectiveness monitoring requirements which take into consideration the type of genetic vector used (chromosomal-integrating or non-integrating), the target cell (stem or differentiated), and the underlying characteristics of the patient population, including the intrinsic risk profile of the disease to be treated [42]. The EMA recommends that safety and efficacy studies for ATMPs use routine clinical practice for follow-up whenever possible to limit additional procedures and interventions for patients [41].

### The challenges of a rare disease registry

Clinicians treating patients with ADA-SCID can currently submit data to disease registries encompassing broader disease classes, such as primary immunodeficiency or SCID, but there are as yet no registries dedicated to ADA-SCID [43, 44]. Indeed, to create a specific registry for ADA-SCID would be an unusual undertaking because of the rarity of the disease and the fact that most patients receive treatment in transplant centres which already participate in large multi-disorder registries. For example, long-term follow-up data from patients with ADA-SCID are included in the transplant procedure registries from EBMT (ProMISe) and ESID (SCETIDE); however, these registries are tailored towards outcomes of HSCT and only collect limited information pertinent to the long-term monitoring of gene therapy. Thus, for Strimvelis, it was necessary to establish a new, observational, patient registry to record long-term longitudinal health data.

A novel approach to the design of the registry was required to address the following barriers to adopting a traditional registry design. The rarity of ADA-SCID and the low number of patients treated with Strimvelis emphasises the need for high patient retention rates in the registry in order to obtain safety and effectiveness data over an extended time frame. The risk of patients being lost to follow-up is exacerbated in a rare disease population formed predominantly of paediatric patients, particularly when monitoring is to be conducted over a minimum 15-year period starting from early infancy. Patients may be lost to follow-up due to movement within/outside their original country arising from progression through schools and on to college in adolescence, or from a family move or changes in the family unit. Children tend to progress through multiple healthcare practitioners (HCPs) as their care is passed between paediatric immunologists, general paediatricians, other specialists and family physicians. Compliance with registry participation may diminish over time if long-term efficacy is achieved and patients start to identify less with their disease. Therefore, long-term follow-up necessitates a commitment on the part of the patient and treating physician to maintain communication links with the registry and to continue to provide ongoing medical information.

Patients from several continents have already been treated with Strimvelis so the follow up system needs to have international coverage. As ADA-SCID is ultra-rare, in many countries there may only be a single child who has received or will receive Strimvelis and who would need to be followed in the registry. Establishing a traditional clinical investigational site with its requisite contracting process, set-up, training and oversight costs would be neither practical nor affordable for such limited numbers of patients per site. For patients who move within/outside their original country, it would not be feasible to establish several investigational sites, potentially contributing to follow-up attrition and missing information. These aspects are both practical and economical, not only affecting the Strimvelis registry, but also gene therapy registries to follow, and any cost efficiency that can be gained from an astute registry design would be expected to encourage further innovation in data collection in the orphan drug space. The objective of developing gene therapy treatments is to create ‘one-off’ interventions. The single treatment model has associated commercial challenges, i.e. recouping research and development costs for commercial sponsors. While it is appropriate that long-term registries are developed to monitor safety and effectiveness of gene therapies, the cost of managing them needs to be controlled such that they do not inhibit innovation.

The aim of this article is to explore the benefits, challenges and overall journey taken to establish a patient-centric registry for this rare disease gene therapy. This registry-based post-authorisation safety study (PASS; EU PAS Registration Number: EUPAS15795) fulfils the EMA guideline for the follow-up of patients administered gene therapy medicinal products.

## Results

### The design of the Strimvelis registry

This registry will enrol a total of 50 patients who received Strimvelis during clinical development, compassionate use and early access programmes, and after marketing authorisation. Patients treated with Strimvelis are eligible to participate in the registry if they (adult patients) or their parents/legal guardians provide informed consent for participation. Each patient will be followed for a minimum of 15 years after treatment with Strimvelis as part of a post-authorisation safety study that will be sponsored by the marketing authorisation holder (MAH) for its duration. Given the extended time frame of the registry, patients will be reconsented as appropriate. Registry closure will occur when the 50th patient has been followed for 15 years. Safety information on any events relating to fertility and pregnancy outcomes, oncogenesis and survival will continue to be solicited every 2 years past a subject’s 15-year post-gene therapy anniversary until the registry is completed. It is anticipated that the majority of subjects enrolled into the registry will be followed-up for longer than 15 years.

Safety concerns evaluated will include, but are not limited to: i) autoimmunity, ii) oncogenesis; presence of replication competent retrovirus (RCR) and retroviral insertion site (RIS) analysis, where these tests have been performed, and iii) medical and surgical procedures associated with Strimvelis administration, including placement and maintenance of central venous catheter and busulfan conditioning (Table 1). Long-term evaluation of safety data from the registry may also provide information relating to immunogenicity, fertility and pregnancy outcomes.

**Table 1:** Efficacy and safety endpoints of the Strimvelis registry post-authorisation safety study

	Baseline data collected from OSR medical records^a^		Collected through standard of care procedures performed by the patient’s local HCP (e.g. referring paediatric immunologist) For patients treated in the clinical studies, baseline and follow-up data will be obtained retrospectively from records at OSR or trial database			
Observational data and minimal time points for their collection	Pre-treatment phase^f^	Treatment phase (Year 0)^f^	Annually Years 1 to 11	Year 13	Year 15	>Year 15
General						
Medical history	✓					
Demographics	✓					
Gene therapy date / dose / lot no.		✓				
Growth (height and weight)	✓		✓	✓	✓	
Survival			✓	✓	✓	✓^g^
Fertility and pregnancy outcomes			✓	✓	✓	✓^g^
Oncogenesis			✓	✓	✓	✓^g^
Development and QoL	✓		✓	✓	✓	
Use of medication/treatments of interest						
ERT, HSCT, radiotherapy, cytotoxic agents			✓	✓	✓	
*AEs*						
Reported SAEs	✓	✓	✓	✓	✓	
Reported AEs	✓	✓	✓	✓	✓	
AEs of interest^a^	✓	✓	✓	✓	✓	
Specialist lab assessments^b^						
dAxP RBCs	✓	✓	✓	✓	✓	
Busulfan AUC		✓				
Immunogenicity^c^		✓	✓	✓	✓	
Vector copy number^d^		✓	✓	✓	✓	
T-cell function	✓	✓	✓	✓	✓	
RIS and RCR^e^			✓	✓	✓	
General lab assessments^b^						
Peripheral lymphocyte counts	✓	✓	✓	✓	✓	
Laboratory blood test results	✓	✓	✓	✓	✓	

*AE* adverse event, *ADA* adenosine deaminase, *AUC* area under the curve, *dAxP* deoxyadenosine nucleotide, *ERT* enzyme replacement therapy, *HCP* healthcare practitioner, *HSCT* haematopoietic stem cell therapy, *OSR* Ospedale San Raffaele, *PEG* polyethylene glycol, *RBC* red blood cell, *RCR* replication competent retrovirus, *RIS* retroviral insertion site, *SAE* serious AE

^a^AEs and SAEs related to medical or surgical procedures associated with Strimvelis administration (e.g., central venous catheter) or related to busulfan conditioning; hypersensitivity (e.g., angioedema, anaphylactic reactions, systemic allergic events and severe cutaneous adverse reactions); autoimmunity, and oncogenesis

^b^When the test is performed as part of standard of care by the treatment centre, local specialist HCP or primary care physician

^c^At baseline, data related to titres of anti-PEG-ADA antibodies, their cross reactivity to human ADA and neutralising activity will be collected. After baseline, data related to titres of anti-ADA antibodies and their neutralising activity will be collected if available

^d^Data collected from assessment during treatment process and when it has been performed by a HCP during follow-up as part of standard of care

^e^Data from RIS analysis and replication competent retrovirus will only be collected if a HCP has performed these tests (e.g. following suspected malignancy or after a diagnosis of malignancy)

^f^Pre-treatment phase: defined as the period from when eligibility for Strimvelis is confirmed in OSR, including when central venous catheter insertion and back-up bone marrow harvest occur, up to the beginning of the Treatment phase. Treatment phase: defined as the period from when the bone marrow harvest for treatment occurs, including conditioning with busulfan and the infusion of transduced CD34+ cells up to and including tests conducted as part of that process

^g^Data on areas of long-term interest (i.e. death, oncogenesis, fertility and pregnancy outcomes) collected every 2 years while the registry is open

Effectiveness will be evaluated by outcomes including survival, intervention-free survival and growth (Table 1). Where patient-reported outcomes are collected during routine medical practice, the registry will capture quality of life (QoL) data collected using validated instruments (PedsQL™ Pediatric Quality of Life Inventory and the Ages and Stages Questionnaire).

### Unique operational features of the Strimvelis registry

The Strimvelis registry has been designed for streamlined data collection and to optimise patient engagement. In order to address the challenges associated with a 15-year follow-up duration in this patient group, in addition to supporting physician data entry, the registry data collection tools focus on and facilitate patient-mediated data entry.

**Fig. 1:**
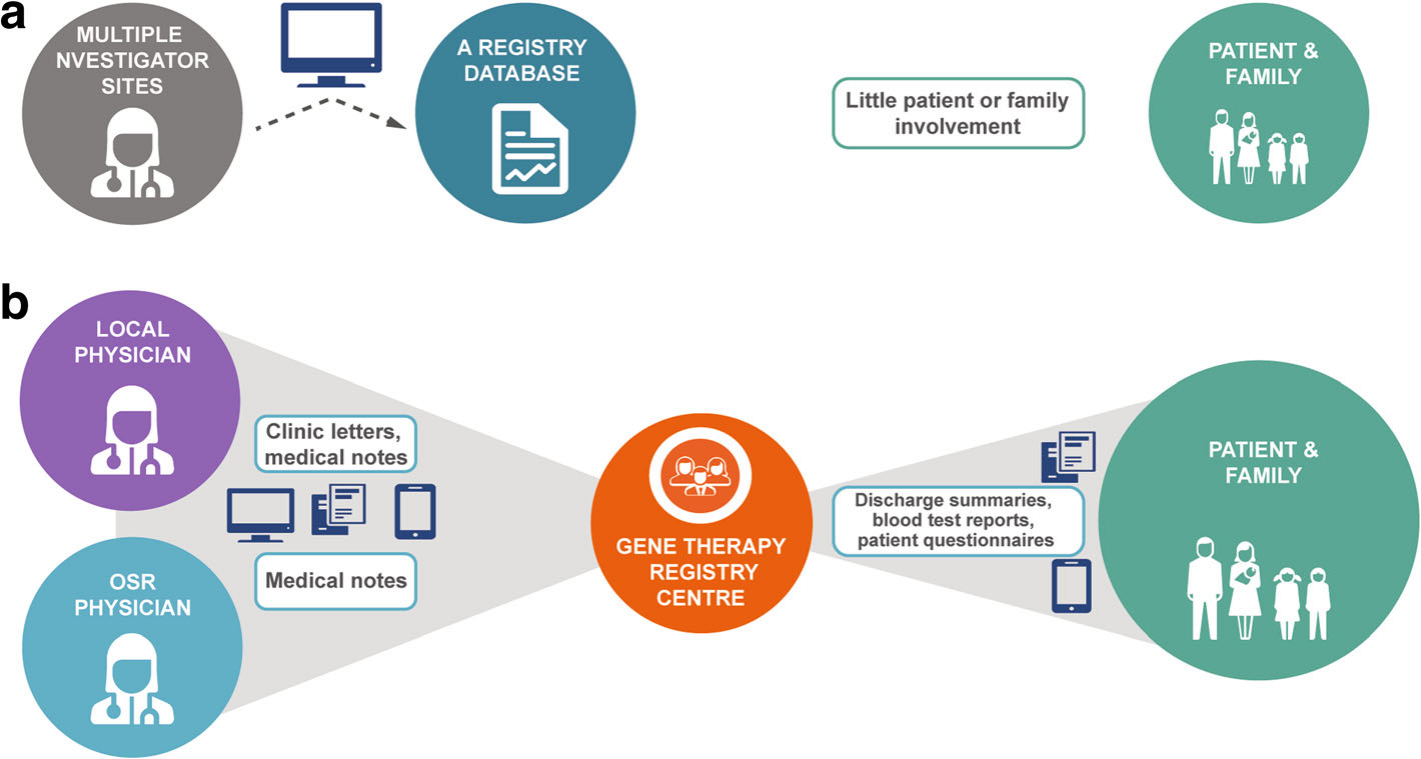
Standard registry model (**a**) and the patient-centric model of the Strimvelis treatment registry (**b**). Both models include standard of care post-treatment follow-up and emergency care. OSR, Ospedale San Raffaele

**Fig. 2:**
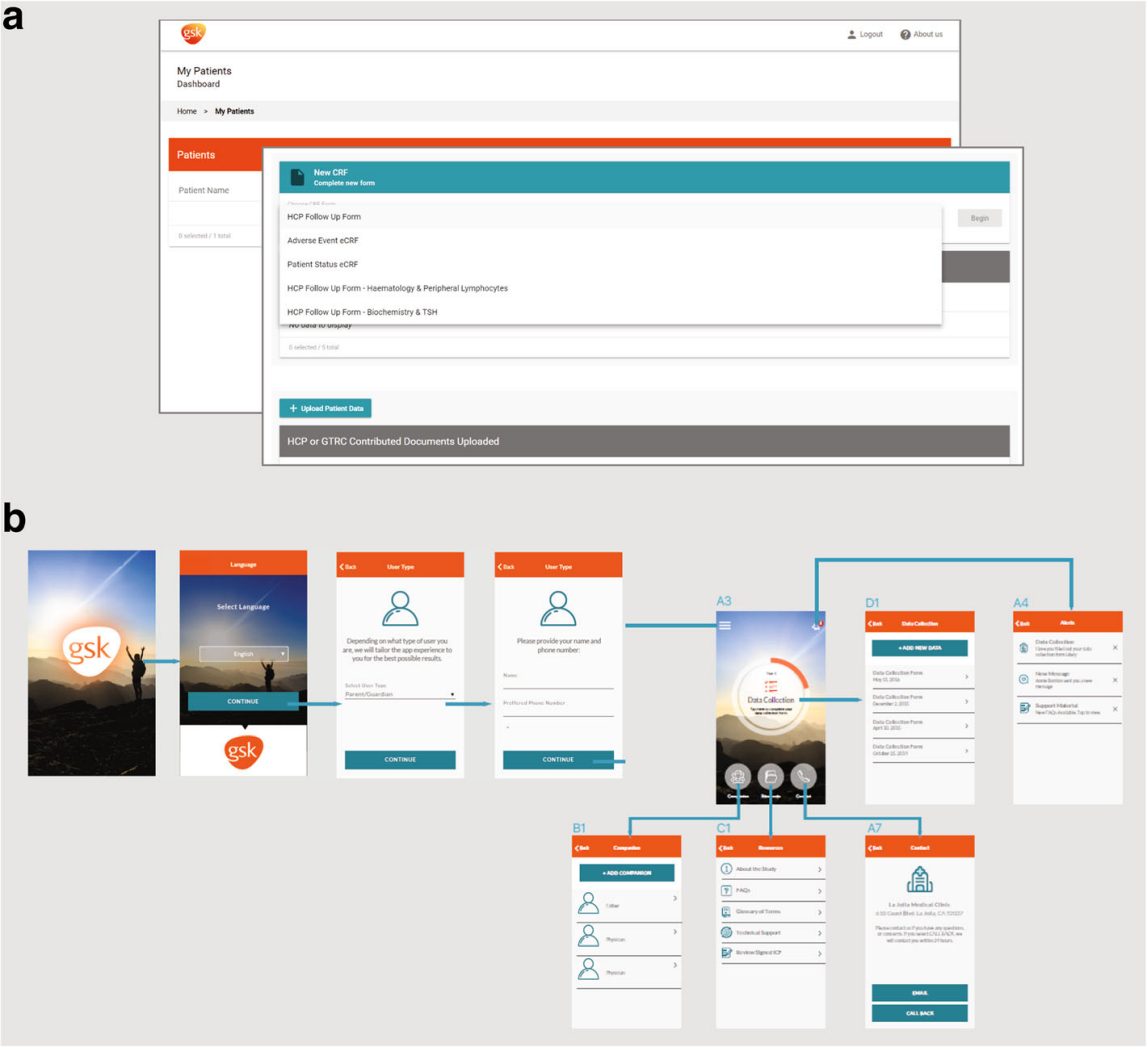
Example physician platform within the Strimvelis registry (**a**) and patient app interface (**b**)

Data will be collated via an electronic platform and coordinated by a gene therapy registry centre (GTRC), run by a contract research organisation (CRO) with local personnel at each country where the patient resides. The intention is to retain a single investigator site at Ospedale San Raffaele (OSR), Milan, Italy, where the treatment was initially developed and is currently administered (Figure 1). Data from OSR will be collected as electronic case report forms (eCRFs), based on information extracted from patient medical records. The patient’s local physician in their country of residence will also be invited to contribute data via an eCRF following permission from patients/families (Figure 2a). In addition, patients and their families will be able to enter data and upload medical records for processing by the GTRC to an electronic registry platform. To enable use by patients, the platform will be prepared in patient-friendly language and will undergo validation and user acceptance testing before release. To encourage patient and family engagement and provide a convenient platform, data input will be facilitated by the use of patients’ own electronic devices. Interactive elements, including a tailored patient app, are included as part of the registry design and will be available for patients to download in multiple languages (Figure 2b).

The GTRC will be operated by trained personnel with the knowledge to ensure data entered by patients/parents/carers is meaningful and accurate. GTRC staff are to maintain regular contact with participating patients/families as part of active follow-up (e.g., quarterly in the first 2 years, annually thereafter until the 11th year and then every 2 years at 13 years, 15 years and beyond), ensuring that patient medical information is collected and flows between the investigator site, the individual patient/parent/carer and the patient’s local HCP (Figure 1). By building relationships with patients and carers, it is anticipated that the inclusion of the GTRC into the registry model will help to mitigate against patients becoming lost to follow-up.

Reporting of an adverse event (AE) to the electronic platform by the local physician (Figure 2a) will trigger a notification to be sent to the GTRC, who will then liaise with the physician as necessary to confirm causality and severity. If an adverse drug reaction (ADR) or serious AE (SAE) is reported, either by the local physician via the GTRC or by the single investigator site (OSR), then the GTRC or OSR will be required to submit the report to the MAH in accordance with protocol-defined time frames, at which point the safety team will follow-up. Other AEs will be added to the database and reviewed periodically. Strimvelis is subject to additional safety monitoring (as indicted by an inverted black triangle symbol), which will encourage patients and HCPs to report AEs. The design of the registry to have a single investigator site (treating centre) will allow for cohesive ongoing monitoring of the safety of Strimvelis as well as detection of disease-related issues (including non-immunologic manifestations) that could arise over time.

### Technical aspects of the Strimvelis registry

The quality of data obtained is of paramount importance to the success of the registry. The design of the registry will need to support patients and their families if they are to provide information with sufficient depth and correct use of medical terms. The GTRC will be integral to this process, maintaining contact with registry participants and performing quality control of inputted data (e.g. manual checks for missing information and inconsistencies and system checks). Query resolution processes, including follow-up with participants where necessary, will help to ensure the integrity of data, and data verification will be sought via HCPs if queries arise. Thoughtful design of user-friendly data entry forms/screens will provide a framework for data input. The GTRC will be available to provide training and guidance to patients and HCPs as required for the duration of the study, in addition to which the platform provider will give technical support. As part of the validation procedure, the MAH or representative will monitor the data provided by OSR to verify, according to the study-specific monitoring plan, that: data are authentic, accurate, and complete; the safety and rights of patients are being protected; and the study is conducted in accordance with the currently approved protocol and any other study agreements, good pharmacovigilance practice, and all applicable regulatory requirements. Representatives of the MAH’s quality assurance unit/monitoring team and regulatory authorities will be permitted to inspect all study-related documents and other materials including but not limited to completed eCRFs and the patients’ original medical records held at OSR. Audits may be conducted at any time during or after the registry at the request of the sponsor or local/international regulators to ensure the validity and integrity of the study data.

Completeness of data is a critical aspect of the registry’s utility, which relies on co-operation of the patient and their HCP. As a divergence from a traditional, investigator-site led PASS, the Strimvelis registry will not establish a formal contract with patients’ HCPs. Instead, HCPs will be asked to acknowledge an agreement via the electronic platform recognising that: the patient/family has provided consent for them to disclose requested information; data they submit should be accurate and complete; they should abide by relevant data privacy provisions, and they agree to ongoing contact with the GTRC. The introduction of GTRC to oversee data collection is expected to aid continued participation from patients and HCPs; however, the nature of data requested from patients and HCPs will have to respect the accepted standard of care and routine clinical practice conducted in each participant’s country of origin to maintain the observational design of the registry. The GTRC will not monitor HCP sites; rather, data quality control will be performed at the level of the single investigator site at OSR.

Data privacy is of paramount importance, and the individual patient profile containing sensitive clinical data must be protected as robustly as possible. In the Strimvelis registry, patients access their individual patient profile in a manner consistent with EU privacy regulations and the platform is built with validated security protections. Platform access is controlled by having predetermined authorised user roles allowing blinded or unblinded access to the data, depending on the user’s specific function. Access to personally identifiable information (PII) will be restricted to OSR and relevant HCPs (e.g. patient’s local physician or specialist), GTRC staff involved in running the registry, and other authorised entities like regulatory agencies, ethics committees or review boards, and persons delegated by the MAH to monitor the registry to make sure it is running properly and that it meets regulatory requirements. All PII will be replaced by code numbers before data are shared with the MAH.

Inevitably, some patients may decide not to participate in the Strimvelis registry or will withdraw or be lost to follow-up. In the case of loss to follow-up or decision to withdraw, all data collected up to that point will be included in the analysis. For patients who complete 15 years of follow-up, the MAH will request permission to continue to collect vital information (e.g. death, oncogenesis, fertility and pregnancy outcomes) every 2 years until registry closure.

## Discussion

The Strimvelis registry represents a departure from the traditional registry model of investigator-led data input. A unique, patient-focused design has been implemented to achieve the retention rates and data completeness required for long-term follow-up of patients treated with gene therapy for a rare disease.

### The value of a combined patient and physician data collection approach

The value of a patient-centric model lies in linking the data with the patient, rather than the clinician, avoiding attrition through changes in HCP [45]. Engaging patients as the main contributors of data circumvents the need for multiple investigator sites while also simplifying the administrative burden on local clinicians. The international ‘borderless’ GTRC-based registry is anticipated to reduce geographical and cultural barriers to registry participation.

A patient-centric model provides motivational factors for patients to stay engaged with the Strimvelis registry, helping to ensure the longevity of registry data collection. Using up to date technology, patients feel supported by GTRC staff, and the adherence to follow-up of the patient/ family may be better if contact with the treating centre via the GTRC is maintained. Patients and their families are encouraged to engage more with their health condition and healthcare by accessing their individual patient profile through the patient app. The collection of patient-reported outcomes through the registry platform considers the patient experience, facilitating a holistic evaluation of the impact of treatment. Participating in a registry for a rare disease helps patients, families and clinicians to feel that they are positively contributing to the knowledge base of sometimes poorly understood and undertreated conditions [45].

Transparency and sharing of data analyses and publications with participants can help to reinforce the kind of information required, and may act to reassure patients of the value of their contributions [45]. Furthermore, communication of research findings in peer-reviewed journal articles and at scientific meetings is seen by the European Platform for Rare Disease Registries (EPIRARE) as a marker of quality in rare disease registries [46].

The value of patient-centric registries is increasingly being recognised in rare disease research. In the USA, the National Organization for Rare Disorders recently launched a National Patient Registry initiative, choosing 20 rare disease patient groups to work with on the development of registries for their patient communities [47]. Through these patient groups, patients and parents/legal guardians of patients are invited to participate in the respective disease-specific registry.

### Sustainability and future development

As well as providing the foundation to evaluate the long-term safety and effectiveness of Strimvelis, the registry has the potential to contribute to the overall understanding of ADA-SCID, providing a more complete overview of outcomes in patients with the disease. To achieve this, the registry would need to evolve to include outcomes from all available patients with ADA-SCID independent of treatment received, rather than remain a single treatment registry. Collaboration with additional partners is currently being sought, in accordance with EU privacy regulations. This approach requires cooperation from all key stakeholders, including clinicians, academics, industry, patient groups and payers. Compatible data sharing between registries would be facilitated by similarities in registry design and the use of common data elements, health indicator values and comparable procedures and definitions during data collection [48]. The benefits of a broader registry are multiple, including determination and comparison of the long-term safety and benefit of different treatments (especially if more treatment options become available), the natural history of the disease, and greater linkage of treatment outcomes to patient characteristics [45]. Identification of patient factors that influence health outcomes in ADA-SCID could lead to treatment algorithms that can be tailored to the individual patient profile or that allow more choice.

## Conclusions

The Strimvelis registry represents a ground-breaking advancement in the long-term pharmacovigilance monitoring of gene therapy treatments. It has been specifically created to evaluate the long-term safety and effectiveness of Strimvelis, the only ex vivo gene therapy approved by the EMA. The Strimvelis registry has been designed for longevity to ensure patient retention by utilising an innovative patient-centric model. By centring data collection with patients and their families, and using interactive elements that take advantage of the convenience of portable electronic devices, the Strimvelis registry aims to maximise patient retention and engagement. These features are expected to address the challenges of long-term follow-up in a paediatric population. In addition, participation in registry data collection is expected to benefit patients, their families and clinicians by helping them to feel that they are positively contributing to the knowledge base of ADA-SCID. Practical and economical efficiencies of the Strimvelis registry, such as an electronic data collection platform facilitating international data collection by a single clinical site, and a mechanism to ensure patient and family engagement over the long term, is expected to serve as a useful model for others developing future therapies for rare diseases. Further evolution of the Strimvelis registry to include outcomes from all available patients with ADA-SCID independent of treatment received, possibly through collaboration with additional partners will allow broader comparisons to be made regarding available treatments for patients with ADA-SCID.

The field of haematopoietic stem cell gene therapy is expanding from its origins in ADA-SCID to include a range of inherited rare diseases [16, 49, 50]. It is therefore highly likely that other autologous stem cell gene therapy treatments will be approved in the future with the added requirement for long-term evaluation of safety and effectiveness. The Strimvelis registry is likely to set a precedent for the long-term pharmacovigilance of future gene therapies.

## Methods

A registry was established to meet the EMA’s regulatory requirements for safety and efficacy monitoring of an approved gene therapy for the treatment of ADA-SCID (see Table 1 for agreed endpoints).

## Abbreviations

ADA: Adenosine deaminase
ADA-SCID: Adenosine deaminase-severe combined immunodeficiency
ADR: Adverse drug reaction
AE: Adverse event
ATMP: Advanced therapy medicinal product
AUC: Area under the curve
CGD: Chronic granulomatous disease
CRO: Contract research organisation
dAxP: Deoxyadenosine nucleotide
EBMT: European Society for Blood and Marrow Transplantation
eCRF: Electronic case report form
EMA: European Medicines Agency
EPIRARE: European platform for rare disease registries
ERT: Enzyme replacement therapy
ESID: European Society for Immunodeficiencies
EU: European Union
GTRC: Gene therapy registry centre
HCP: Healthcare professional
HSCT: Haematopoietic stem cell transplantation
MAH: Marketing authorisation holder
MFD: Matched family donor
MSD: Matched sibling donor
OSR: Ospedale San Raffaele
PASS: Post-authorisation safety study
PEG: Polyethylene glycol
PII: personally identifiable information
QoL: Quality of life
RBC: Red blood cell
RCR: Replication competent retrovirus
RIS: Retroviral insertion site
SAE: Serious adverse event
T-ALL: T-cell acute lymphoblastic leukaemia
US: United States
WAS: Wiskott−Aldrich syndrome
X-SCID: X-linked severe combined immunodeficiency
